# Evaluation of prognostic markers for canine mast cell tumors treated with vinblastine and prednisone

**DOI:** 10.1186/1746-6148-4-32

**Published:** 2008-08-13

**Authors:** Joshua D Webster, Vilma Yuzbasiyan-Gurkan, Douglas H Thamm, Elizabeth Hamilton, Matti Kiupel

**Affiliations:** 1 Comparative Medicine and Integrative Biology Program, College of Veterinary Medicine, Michigan State University, East Lansing, MI, USA; 2 Department of Pathobiology and Diagnostic Investigation, College of Veterinary Medicine, Michigan State University, East Lansing, MI, USA; 3 Department of Small Animal Clinical Sciences, College of Veterinary Medicine, Michigan State University, East Lansing, MI, USA; 4 Department of Microbiology and Molecular Genetics, College of Veterinary Medicine, Michigan State University, East Lansing, MI, USA; 5 Animal Cancer Center, College of Veterinary Medicine and Biomedical Sciences, Colorado State University, Fort Collins, CO, USA

## Abstract

**Background:**

Canine cutaneous mast cell tumor (MCT) is a common neoplastic disease associated with a variable biologic behavior. Surgery remains the primary treatment for canine MCT; however, radiation therapy (RT) and chemotherapy are commonly used to treat aggressive MCT. The goals of this study were to evaluate the prognostic utility of histologic grade, *c-KIT *mutations, KIT staining patterns, and the proliferation markers Ki67 and AgNORs in dogs postoperatively treated with vinblastine and prednisone +/- RT, and to compare the outcome of dogs treated with post-operative chemotherapy +/- RT to that of a prognostically matched group treated with surgery alone. Associations between prognostic markers and survival were evaluated. Disease-free intervals (DFI) and overall survival times (OS) of dogs with similar pretreatment prognostic indices postoperatively treated with chemotherapy were compared to dogs treated with surgery alone.

**Results:**

Histologic grade 3 MCTs, MCTs with c-*KIT *mutations, MCTs with increased cytoplasmic KIT, and MCTs with increased Ki67 and AgNOR values were associated with decreased DFI and OS. Dogs with histologic grade 3 MCT had significantly increased DFI and OS when treated with chemotherapy vs. surgery alone. Although not statistically significant due to small sample sizes, MCTs with *c-KIT *mutations had increased DFI and OS when treated with chemotherapy vs. surgery alone.

**Conclusion and clinical importance:**

This study confirms the prognostic value of histologic grade, c-*KIT *mutations, KIT staining patterns, and proliferation analyses for canine MCT. Additionally, the results of this study further define the benefit of postoperative vinblastine and prednisone for histologic grade 3 MCTs.

## Background

Canine cutaneous mast cell tumor (MCT) is a common neoplastic disease in dogs, accounting for 7–21% of all cutaneous neoplasms [1-4]. Canine MCTs have variable biologic behaviors, ranging from solitary benign masses that can be cured with surgery alone to systemic and potentially fatal metastatic disease [5-9]. Currently, surgical excision is the primary treatment modality for canine MCT. In the event of incomplete surgical excision or a non-resectable tumor, radiation therapy (RT) may be used as an adjunct local therapy, and in the face of multicentric, metastatic, or aggressive MCT, postoperative chemotherapy is commonly employed. Canine mast cell tumors have variable response rates to different chemotherapy protocols, and, as with any drug, there is potential for the development of serious adverse events as a result of chemotherapy administration[6,8]. Therefore, it is critical to identify patients that will benefit most from such treatments and to identify prognostic markers that are associated with specific treatment outcomes.

Previously, our laboratory has evaluated the prognostic significance of several markers for canine MCT patients treated with surgery alone. In these studies we found that KIT staining patterns[10], *c-KIT *internal tandem duplication (ITD) mutations[11], and cellular proliferation as measured by Ki67 immuno-staining and AgNOR histochemical staining[12] are significantly associated with the progression of canine MCT when treated with surgery alone. Although the results of these studies demonstrate the utility of these markers as diagnostic and prognostic tools for MCT treated with surgery alone, they do not provide information regarding their association with the outcome of patients treated with postoperative RT or chemotherapy.

Combination chemotherapy with vinblastine and prednisone has been previously suggested to be an efficacious postoperative therapy for a subset of MCT patients[13]; however, no clear determinants have been identified to discriminate between MCTs that are likely to benefit from vinblastine and prednisone and those that are not. Therefore, the primary goal of this study was to evaluate the prognostic significance of histologic grade; *c-KIT *ITD mutation; KIT staining patterns; and the proliferation markers Ki67 and AgNORs for dogs treated with vinblastine and prednisone following surgery +/- RT. Additionally, in order to better determine the efficacy of postoperative vinblastine and prednisone, an additional goal of this study was to compare the outcomes of dogs postoperatively treated with vinblastine and prednisone +/- RT to those treated with surgery alone, when stratified based on identified prognostic markers. The results of this paper show that histologic grade; *c-KIT *ITD mutation; KIT staining patterns; and the proliferation markers Ki67 and AgNORs are prognostically significant for dogs with MCTs treated with vinblastine and prednisone following surgery +/- RT. Additionally, this study strongly suggests that dogs with grade 3 MCTs benefit from post-operative chemotherapy treatment when compared to treatment with surgery alone.

## Results

Twenty-eight MCTs dogs postoperatively treated with vinblastine and prednisone +/- RT were included in this study. Fourteen dogs were female and 14 were male. Dogs ranged from 2 to 14 years of age (mean = 9.29 years) and represented 16 breeds including mixed breed dogs (9), Labrador retrievers (4), Gordon setters (2), and 13 other breeds which were represented by single animals. Ten tumors were histologic grade 3 MCT and 18 were grade 2. Four MCTs, all of which were histologic grade 3 tumors, had ITD *c-KIT *mutations. Two MCTs had pattern 1 KIT protein expression, 6 MCTs had KIT pattern 3, and 20 MCTs had KIT pattern 2.

**Figure 1:**
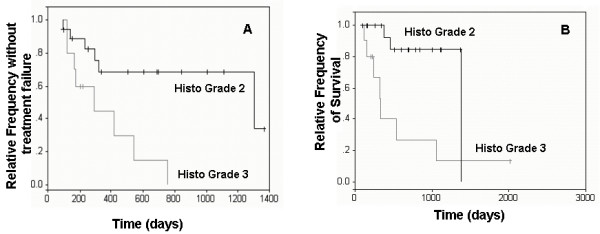
**Survival curves of dogs with histologic grade 2 and 3 MCTs**. Kaplan-Meier survival curves comparing disease-free interval (A) and overall survival times (B) of dogs with histologic grade 2 and 3 MCTs.

**Figure 2:**
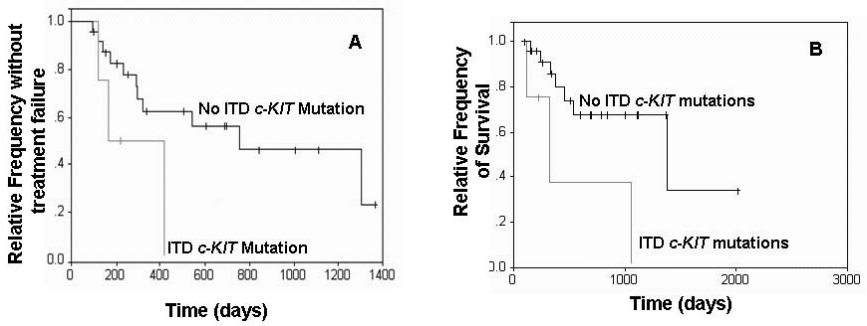
**Survival curves of dogs with and without *c-KIT *mutations**. Kaplan-Meier survival curves comparing disease-free interval (A) and overall survival times (B) of dogs with and without *c-KIT *mutations.

**Figure 3:**
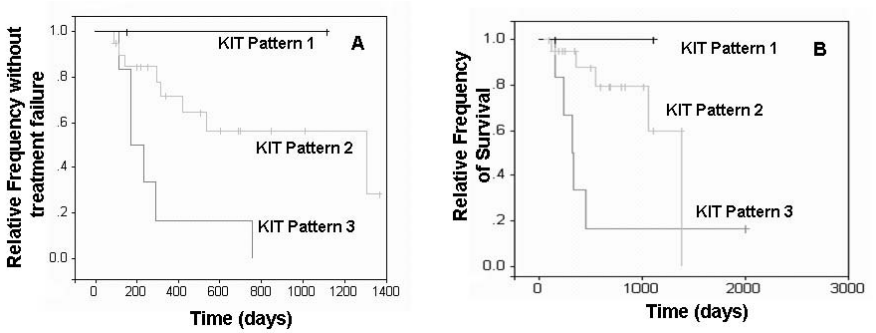
**Survival curves of dogs with different KIT staining patterns**. Kaplan-Meier survival curves comparing disease-free interval (A) and overall survival times (B) of dogs with MCTs with different KIT staining patterns.

Results of multivariable analyses are presented in Table 1. According to multivariable analyses, dogs with histologic grade 3 MCT and dogs with ITD *c-KIT *mutations had significantly shorter disease-free intervals (DFI) (p = 0.0220 and p = 0.0413, respectively) and overall survival times (OS) (p = 0.010 and p = 0.0112, respectively) compared to dogs with grade 2 MCT and dogs lacking *c-KIT *mutations, respectively (Figures 1 and 2). Tumors with KIT staining pattern 3 were significantly associated with decreased DFI and OS compared to tumors with KIT pattern 2 (p = 0.0022 and p = 0.0049, respectively; Figure 3). Increased Ki67 expression and AgNOR values were associated with decreased DFI (p = 0.0386 and p = 0.0168, respectively) and decreased OS (p = 0.0097 and p = 0.0066, respectively) [12].

**Table 1:** Multivariate analyses of associations between prognostic factors and patient outcome.

**Variable**	**N**	Median DFI*	**DFI p-value**	**Median OS time^**# **^**	**OS time p-value**
		**(months)**		**(months)**	
Histologic grade					
*Grade 2*	18	14.1	0.022	21.5	0.01
*Grade 3*	10	7		9.2	
KIT staining pattern^+^					
*Pattern 2*	20	12.7	0.0022	19	0.0049
*Pattern 3*	6	6.7		10.8	
*c-KIT *mutation					
*No*	24	11	0.0413	17.4	0.0112
*Yes*	4	6.5		8.9	

*DFI: disease-free interval.

^#^OS: overall survival.

^+^Two KIT pattern 1 MCTs were excluded from statistical analyses.

Although previous studies have described patient survival following postoperative treatment with vinblastine and prednisone[13], no studies have compared the outcome of treatment with vinblastine and prednisone to that of surgery alone in a similar group of dogs. In order to retrospectively evaluate the efficacy of postoperative vinblastine and prednisone, survival curves representing DFI and OS times were compared between dogs treated in this study and dogs treated with surgery alone that were evaluated as part of previous studies[10-12,14]. As a means of ensuring that similar populations of animals were compared, comparisons were made between groups of animals that had similar pre-treatment prognoses based on histologic grade, KIT staining pattern, and the presence of ITD *c-KIT *mutations.

**Figure 4:**
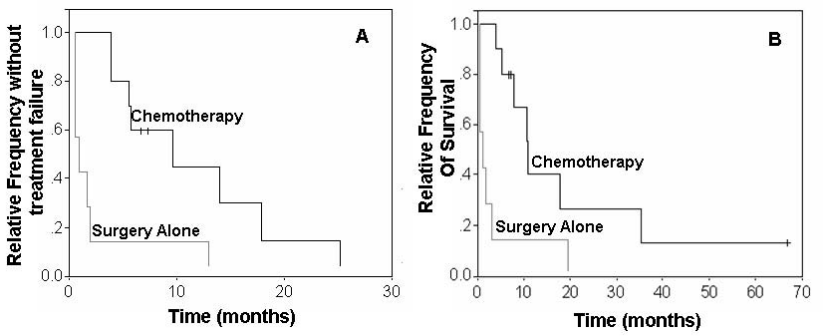
**Survival curves of dogs with grade 3 MCTs treated with surgery alone or with post-operative chemotherapy**. Kaplan-Meier survival curves comparing disease-free interval (A) and overall survival times (B) of dogs with histologic grade 3 MCT treated with surgery alone or with vinblastine and prednisone in addition to surgery.

**Figure 5:**
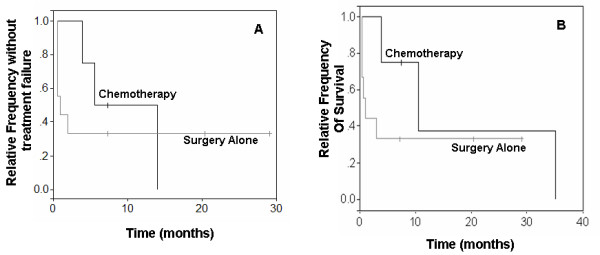
**Survival curves of dogs with *c-KIT *mutations treated with surgery alone or with post-operative chemotherapy**. Kaplan-Meier survival curves comparing disease-free intervals (A) and overall survival times (B) of dogs with *c-KIT *mutations treated with surgery alone or with vinblastine and prednisone in addition to surgery.

According to multivariable analyses, dogs with histologic grade III MCT treated with surgery alone had significantly shorter DFI (1 month vs. 7 months, p = 0.0082) and OS times (1 month vs. 9.2 months, p = 0.0427) compared to dogs treated with vinblastine and prednisone (Figure 4). Dogs with KIT pattern 2 MCT treated with surgery alone had significantly shorter OS times compared to those treated with vinblastine and prednisone (16.6 months vs. 19 months, p = 0.0362). Mast cell tumors with c-*KIT *mutations treated with vinblastine and prednisone tended to have increased DFI (1 month vs. 6.5 months) and OS times (1 month vs. 8.9 months); however, these associations were not statistically significant (Figure 5).

## Discussion

In this study, treatment with vinblastine and prednisone following surgery +/- RT benefited dogs with grade III MCT over treatment with surgery alone. The results of this study further validate the use of postoperative vinblastine and prednisone for histologic grade III MCT. Additionally, dogs with *c-KIT *mutations that were treated with vinblastine and prednisone tended to have longer DFI and OS times as compared to those treated with surgery alone, although this association was not statistically significant. This lack of statistical significance might be due to the relatively low number of cases with *c-KIT *mutations evaluated or the similar relative frequency of DFI and OS after 5 and 10 months, respectively. Additionally, 2/9 dogs with *c-KIT *mutations treated with surgery alone survived greater than 20 months. However, one mutation was confined to intron 11; therefore the biologic significance of this mutation is unknown[11]. The difference in median OS times between MCTs with *c-KIT *mutations treated with local therapy alone and those treated with vinblastine and prednisone suggests that MCTs with *c-KIT *mutations also might benefit from postoperative chemotherapy with vinblastine and prednisone.

In a previous study, we showed MCTs treated with surgery alone with more than 23 Ki67-positive cells per grid area or a Ki67 × AgNOR (Ag67) value of more than 53 per grid area were associated with decreased patient survival[12]; however, in this study, these cut-off values did not discriminate patient survival. Interestingly, in this study only 2/14 dogs with Ki67 indices < 23 died by the end of this study, while 8/14 dogs with Ki67 indices > 23 died. Similarly only 1/14 dogs with Ag67 indices < 53 died, while 9/14 dogs with Ag67 indices > 53 died by the end of this study. The lack of statistical significance in this study is likely due to variations in the patient populations of these studies and the small population size in this study. Our previous study included 8 dogs with histologic grade 1 MCT, while this study only included grade 2 and grade 3 MCTs. Additionally, all cases included in this study received chemotherapy, suggesting that as a whole, this study was biased towards more aggressive MCT.

In this study, histologic grade, the presence of *c-KIT *mutations, KIT staining patterns, and proliferation markers, Ki67 and AgNORs, were associated with the prognosis of MCT postoperatively treated with vinblastine and prednisone. Compared with grade 2 MCTs, histologic grade 3 MCTs were significantly associated with decreased DFI and OS times. Additionally, dogs with grade 3 MCT significantly benefited from treatment with vinblastine and prednisone. These results support the use of histologic grading for the prognostication of canine MCT; however, interobserver variations and the unpredictable behavior of grade 2 MCT continue to limit the utility of histologic evaluation as the sole predictor of outcome [15-19]. Therefore, in the face of intermediate grade MCTs, histologic grade should be complemented with additional prognostic markers, such as KIT immunohistochemistry, *c-KIT *mutation evaluation, and proliferation markers using Ki67 and Ag67 cut-off values of 23 and 53, respectively. No single marker can successfully identify all MCTs that will result in death; therefore, a panel of markers should be used in order to evaluate the entire spectrum of the disease.

Major limitations of this study include the levels of historical inaccuracy associated with any retrospective study, small samples sizes in some subgroups, and variations in treatment and staging; most notably the fact that 50% of the chemotherapy treated patients received RT. However, dogs treated with chemotherapy and radiation therapy did not have significantly different times to treatment failure or overall survival times as compared to those treated without radiation therapy (log rank p-value = 0.223 and p = 0.314, respectively). An additional limitation of this study was that many of the control treated animals were treated in primary care private practices, while those treated with chemotherapy were treated at a single referral practice. Differences in care between clinicians and hospitals might further affect treatment outcomes; however, data available from for this study do not allow us to evaluate this effect.

## Conclusion

The results of this study add further credence supporting the use of histologic grade, *c-KIT *mutations, and KIT protein localization for the prognostication of canine MCTs. Additionally, these results further support that hypothesis that *c-KIT *mutations and intracellular localization of the KIT protein both contribute to the progression of canine MCT. Finally, although previous studies have evaluated the use of vinblastine and prednisone for the treatment of high-risk MCTs, this is the first study to compare the outcome of animals treated with adjuvant vinblastine and prednisone +/- RT to a prognostically matched group of animals treated with surgery alone. Although retrospective studies provide critical, relevant information regarding the utility of treatments and prognostic markers, controlled, blinded prospective studies remain the gold standard for evaluating therapeutics and prognostic markers and the data presented here need to be corroborated in a controlled, prospective study in order to truly define their prognostic significance. In the future, prospective evaluations of new therapeutics should not only be focused on the safety and efficacy of the therapy, but also on identifying biomarkers including histopathology, clinical pathology, and molecular biology-based markers associated with outcome.

## Methods

Twenty-eight canine cutaneous MCTs from 28 dogs included in this study were identified as part of a larger retrospective study[13] with the goals of: 1. evaluating the combination of vinblastine and prednisone as postoperative chemotherapy for canine MCT following surgical excision +/- RT; and 2. identifying clinical prognostic factors associated with this treatment. All cases were treated at the University of Wisconsin – Madison Veterinary Medical Teaching Hospital and were included in this study based on the following inclusion criteria: 1. absence of severe concurrent disease; 2. complete staging; 3. no concurrent systemic antineoplastic therapy other then prednisone and vinblastine; 4. absence of measurable disease following surgery; 5. confirmed histologic diagnosis of canine cutaneous MCT; 6. adequate tissues available for *c-KIT *polymerase chain reaction and immunohistochemistry. Prednisone was administered at an initial dose of 2 mg/kg PO daily, and tapered over 12–26 weeks. Vinblastine was administered at 2 mg/m^2 ^as a rapid intravenous bolus weekly for 4 weeks, followed by 4 biweekly treatments. One dog was given a total of 16 vinblastine treatments, as treatment was re-initiated at the time of disease recurrence. Tumors were considered to have been treated with "adequate local therapy" when tumors were completely excised with no evidence of neoplastic mast cells at the surgical margins or when surgical excision was performed with subsequent local RT. According to these standards, adequate local therapy was obtained in 24 of 28 dogs. Four dogs had histologic evidence of microscopic disease at the surgical site at the time that chemotherapy was initiated. Three of these dogs had histologic grade 2 MCT and one dog had grade 3 MCT. Postoperative RT was performed in 14 patients, and regional lymph nodes were irradiated in 10 of these patients. Eleven dogs received 4 once-weekly, 8 Gray radiation treatments; one dog received 4 once weekly, 6.5 Gray treatments; and 2 dogs received 15 daily (M-F) treatments of 3.2 Gray each. Histologic grade of each tumor was confirmed according to the Patnaik histologic grading system for canine cutaneous MCT by a single pathologist[7]. Complete clinical staging was performed in all patients, and included physical examination, complete blood count, serum biochemistry profile, abdominal ultrasound, and regional lymph node palpation with or without fine needle aspiration cytology. Lymph nodes were only considered positive for lymph node metastasis if they contained clusters or sheets of mast cells; scattered individual mast cells were not sufficient to diagnose lymph node metastasis. A control population of 56 dogs with canine MCT treated with surgery alone, which were evaluated as part of a previous study[12], were used to compare outcomes between dogs treated with surgery alone and those treated with surgery and postoperative chemotherapy. These cases were submitted as routine biopsies to the Diagnostic Center for Population and Animal Health from multiple referring veterinarians; therefore, clinical staging was not standardized among these cases. Of these 56 dogs, 8 dogs had histologic grade 1 MCT, 41 dogs had grade 2 MCT, and 7 dogs had grade 3 MCT. Nine of these dogs had internal tandem duplication *c-KIT *mutations.

Immunohistochemical staining, AgNOR staining; evaluation of KIT staining patterns, evaluation of proliferation indices as measured by Ki67 labeling and AgNOR staining, and analysis of *c-KIT *mutations were performed as previously described[12] In brief, 3 patterns of KIT protein localization were identified: 1. KIT pattern I, which consisted of a predominately peri-membrane pattern of KIT protein localization with minimal cytoplasmic KIT protein localization; 2. KIT pattern II, which consisted of focal to stippled cytoplasmic KIT protein localization; and 3. KIT pattern III, which consisted of diffuse KIT cytoplasmic KIT protein localization. Each MCT was classified based on the highest staining pattern present in at least 10% (estimated based on 100 neoplastic cells in a high power field) of the neoplastic cell population or being present in large clusters. For Ki67 immunohistochemical staining evaluations, areas with the highest proportion of immuno-positive neoplastic mast cells were identified at 100× magnification using an American Optical light microscope. The number of immuno-positive cells present in a 10 mm × 10 mm grid area was counted using a 1 cm^2 ^10 × 10 grid reticle at 400× magnification. The number of immuno-positive cells per grid area was evaluated in 5 high powered fields and subsequently averaged in order to obtain the growth fraction. In order to determine the average AgNOR count/cell in each tumor, AgNORs were counted in 100 randomly selected neoplastic mast cells throughout the tumor at 1000× magnification. Individual AgNORs were resolved by focusing up and down while counting within individual nuclei. Average AgNOR counts/cell was determined based on averaging these counts.

### Statistical Analysis

This study used two different approaches to analysis: log-rank models(SAS PROC LIFETEST) to describe associations between risk factors and the occurrence (yes/no) of MCT outcomes, and the Cox proportional hazards models (SAS PROC PHREG)^b ^for survival analysis to describe the relationships between risk factors and the time to the occurrence of different MCT outcomes (time-to-event). Survival analysis produces point estimates of the hazard ratio (risk ratio) for risk factors in the model. The MCT outcomes used in this study included disease-free interval and overall survival time. Median times for disease-free interval (DFI) and overall survival (OS) times were reported as the time from treatment initiation to event or last follow-up. Disease-free interval was defined as the time until local recurrence, development of metastatic disease, or development of a new mast cell tumor.

#### Univariable Analyses

Before developing multivariable models, each risk factor was evaluated for its association with MCT outcomes. Both univariable log-rank (for occurrence) and proportional hazards (for time to event) models were developed for each risk factor for each outcome, and the level of association was assessed through the risk factor's p-value in the model. Risk factors with p less than or equal to 0.20 were considered for inclusion in the multivariable models.

#### Multivariable Survival Analysis Models

Multivariable proportional hazards regression models were developed for survival analysis of different outcomes associated with MCT, DFI and OS. As for the multivariable log-rank models, animal signalment (age, sex, weight) was included in the multivariable models to account for their effects on model outcomes. The effects of risk factors on days to events were reported as p-values. The cut off for significance of the multivariate analysis was set a p < 0.05.

## Abbreviations

Disease-free intervals: DFI; mast cell tumor: MCT; overall survival times: OS; radiation therapy: RT.

## Authors' contributions

JDW coordinated the study, performed histologic and immunohistologic evaluations, performed polymerase chain reactions, and drafted the manuscript. VY–G and MK designed the study and oversaw all post-clinical aspects of the study. EH performed all statistical analyses. DHT collected all clinical data. All authors helped interpret results and have read and approved the final manuscript.

## References

[B1] Brodey RS (1970). Canine and feline neoplasia. Adv Vet Sci Comp Med.

[B2] Finnie JW, Bostock DE (1979). Skin neoplasia in dogs. Aust Vet J.

[B3] Priester WA (1973). Skin tumors in domestic animals. Data from 12 United States and Canadian colleges of veterinary medicine. J Natl Cancer Inst.

[B4] Rothwell TL, Howlett CR, Middleton DJ, Griffiths DA, Duff BC (1987). Skin neoplasms of dogs in Sydney. Aust Vet J.

[B5] Bostock DE (1973). The prognosis following surgical removal of mastocytomas in dogs. J Small Anim Pract.

[B6] Misdorp W (2004). Mast cells and canine mast cell tumours. A review. Vet Q.

[B7] Patnaik AK, Ehler WJ, MacEwen EG (1984). Canine cutaneous mast cell tumor: morphologic grading and survival time in 83 dogs. Vet Pathol.

[B8] Thamm DH, Vail DM, Withrow SJ, MacEwen EG (2001). Mast cell tumors. Small Animal Clinical Oncology.

[B9] Zemke D, Yamini B, Yuzbasiyan-Gurkan V (2002). Mutations in the juxtamembrane domain of c-KIT are associated with higher grade mast cell tumors in dogs. Vet Pathol.

[B10] Webster JD, Kiupel M, Kaneene JB, Miller R, Yuzbasiyan-Gurkan V (2004). The use of KIT and tryptase expression patterns as prognostic tools for canine cutaneous mast cell tumors. Vet Pathol.

[B11] Webster JD, Yuzbasiyan-Gurkan V, Kaneene JB, Miller R, Resau JA, Kiupel M (2006). The role of c-KIT in tumorigenesis: evaluation in canine cutaneous mast cell tumors. Neoplasia.

[B12] Webster JD, Yuzbasiyan-Gurkan V, Miller RA, Kaneene JB, Kiupel M (2007). Cellular Proliferation in Canine Cutaneous Mast Cell Tumors: Associations with c-KIT and Its Role in Prognostication. Vet Pathol.

[B13] Thamm DH, Mauldin EA, Vail DM (1999). Prednisone and vinblastine chemotherapy for canine mast cell tumor--41 cases (1992-1997). J Vet Intern Med.

[B14] Kiupel M, Webster JD, Miller RA, Kaneene JB (2005). Impact of tumour depth, tumour location and multiple synchronous masses on the prognosis of canine cutaneous mast cell tumours. J Vet Med A Physiol Pathol Clin Med.

[B15] Kiupel M, Webster JD, Bailey KL, Best S, DeLay J, Detrisac CJ, Gamble D, Ginn PE, Goldschmidt MH, Hendrick MJ, Howerth EW, Janovitz EB, Lenz SD, Lipscomb T, Miller E, Misdorp W, Moroff S, Neyens I, O'Toole D, Ramos-Vara J, Scase TJ, Schulman Y, Smith K, Snyder P, Southorn E, Stedman NL, Steficek BA, Stromberg PC, Valli VE, Weisbrode SE, Yager J, Kaneene JB (2004). Microscopic grading of canine cutaneous mast cell tumors: a multi-institutional review. Vet Pathol.

[B16] Northrup NC, Harmon BG, Gieger TL, Brown CA, Carmichael KP, Garcia A, Latimer KS, Munday JS, Rakich PM, Richey LJ, Stedman NL, Cheng AL, Howerth EW (2005). Variation among pathologists in histologic grading of canine cutaneous mast cell tumors. J Vet Diagn Invest.

[B17] Northrup NC, Howerth EW, Harmon BG, Brown CA, Carmicheal KP, Garcia AP, Latimer KS, Munday JS, Rakich PM, Richey LJ, Stedman NL, Gieger TL (2005). Variation among pathologists in the histologic grading of canine cutaneous mast cell tumors with uniform use of a single grading reference. J Vet Diagn Invest.

[B18] Seguin B, Leibman NF, Bregazzi VS, Ogilvie GK, Powers BE, Dernell WS, Fettman MJ, Withrow SJ (2001). Clinical outcome of dogs with grade-II mast cell tumors treated with surgery alone: 55 cases (1996-1999). J Am Vet Med Assoc.

[B19] Weisse C, Shofer FS, Sorenmo K (2002). Recurrence rates and sites for grade II canine cutaneous mast cell tumors following complete surgical excision. J Am Anim Hosp Assoc.

